# Chief complaints and computed tomography results in the emergency department: a three-year retrospective cohort study

**DOI:** 10.1186/s12873-024-01003-z

**Published:** 2024-05-20

**Authors:** Cheng-Yi Fan, Chi-Hsin Chen, Jiun-Wei Chen, Jia-How Chang, Edward Pei-Chuan Huang, Chih-Wei Sung

**Affiliations:** 1 Department of Emergency Medicine, National Taiwan University Hospital Hsin-Chu Branch, Hsinchu, 300 Taiwan; 2 Department of Emergency Medicine, National Taiwan University Hospital, Taipei, 100 Taiwan; 3 Department of Emergency Medicine, College of Medicine, National Taiwan University, Taipei, 100 Taiwan

**Keywords:** Computed tomography, Chief complaint, Revisit, Emergency department, System-based symptoms

## Abstract

**Background:**

Computed tomography (CT) is frequently performed in the patients who admitted to the emergency department (ED), discharged but returned to ED within 72 h. It is unknown whether the main complaints of patients assist physicians to use CT effectively. This study aimed to find the association between chief complaints and the CT results.

**Methods:**

This three-year retrospective cohort study was conducted in the ED of a tertiary medical center. Adult patients who returned to the ED after the index visit were included from 2019 to 2021. Demographics, pre-existing diseases, chief complaints, and CT region were recorded by independent ED physicians. A logistic regression model with an odds ratio (OR) and 95% confidence interval (CI) was used to determine the relationship between chief complaints and positive CT results.

**Results:**

In total, 7,699 patients revisited ED after the index visit; 1,202 (15.6%) received CT. The top chief complaints in patients who received CT were abdominal pain, dizziness, and muscle weakness. Patients with abdominal pain or gastrointestinal symptoms had a significantly higher rate of positive abdominopelvic CT than those without it (OR 2.83, 95% CI 1.98–4.05, *p* < 0.001), while the central nervous system and cardiopulmonary chief complaints were not associated (or negatively associated) with new positive CT findings.

**Conclusion:**

Chief complaints of patients on revisit to the ED are associated with different yields of new findings when CT scans of the chest, abdomen and head are performed. Physicians should consider these differential likelihoods of new positive findings based on these data.

**Supplementary Information:**

The online version contains supplementary material available at 10.1186/s12873-024-01003-z.

## Introduction

Revisit to the emergency department (ED) within 72 h of index visit discharge is an important issue for ED patient care. The etiology of revisit can vary. The patient may have a higher risk for disease progression, increased complications in hospital or longer length of stay if the cause of the review is associated with a potential misdiagnosis in the ED index visits [1–3]. Clinically, physicians tend to perform more comprehensive examinations on patients following revisits, including detailed image modality, particularly computed tomography (CT) [4].

The use of CT may increase the diagnostic efficiency in some diseases or medical conditions, such as pulmonary embolism [5], acute appendicitis and traumatic brain injury [6], especially in patients with ED revisit. However, to administer CT should be made with caution because the unrestrained use may increase medical cost, radiation exposure, and iatrogenic contrast medium toxicity [7].

Chief complaints, the first pieces of information that ED physicians obtain from patients, may be an alternative indicator for wisely administering CTs. Chief complaints inform focused history taking, physical examination, and treatment priority. Next, blood tests and imaging modalities confirm a tentative diagnosis. Chief complaints in the ED are associated with outcomes such as hospitalization, intensive care unit admission, ED revisits, in-hospital mortality, and long-term mortality [8, 9]. Little is known about the association between chief complaints and CT findings.

Since patients who returned to ED are vulnerable to disease progression or undiagnosed diseases, chief complaint-based CT administration should be considered. Whether chief complaints reflect the CT result remains unknown. This study aimed to investigate the association between chief complaints from patients at revisit and the result of the subsequent CT examinations.

## Method

### Study design and setting

This single-center retrospective cohort study was conducted between January 2019 and December 2021 in the ED of a tertiary medical center with over 800 beds and 1,700 staff. The ED had approximately 60,000 annual visits during the period. There were 20 physicians on staff who covered the emergency department.

This study was approved by the Institutional Review Board of the National Taiwan University, Hsin-Chu Hospital (No. 109-003-E). Research was carried out through reviews of electronic medical records without any medical intervention. Informed consent was therefore waived. Patients or the public were not involved in the design, conduct, reporting, or dissemination plans of our research.

### Patient selection

Patient selection was based on the electronic medical database that contains medical charts and ED triage information. Patients who had a repeat visit within 72 h were included if they were over 20 years old and received a CT on the revisits. Patients were excluded if they left the ED against medical advice or if their medical records were missing. The enrolled patients were divided into three groups by CT imaging region: head, chest, and abdomen. If patients received CT in multiple regions, they were included in the group for each region in which they received CT.

### Data collection and variables

To obtain the correct medical records, this study collected data directly from manually reviewed electronic medical records rather than exporting data from an integrated medical database. Any ED physician who were blinded to the study design and hypothesis was trained to review medical records. Additionally, they received a predefined data coding book that included variable definitions, the coding rules of the binary and categorical variable coding rules and missing variable management. The review process referred to recommendations from chart review studies [10, 11] and was discussed at periodic study meetings. Variables were collected from demographics, pre-existing medical conditions, chief complaints, examinations and treatments on the first visit and revisit, and CT reports on the revisit. Demographics included age and sex. Pre-existing medical conditions, such as hypertension, diabetes mellitus, previous stroke, coronary artery disease, chronic kidney failure, and cancer, were extracted and double-confirmed with outpatient charts and regular medication. We selected the three to five chief complaints recorded on our medical charts from three systems: central nervous, cardiopulmonary, and gastrointestinal and urological systems. Symptoms associated with the central nervous system included headache, dizziness, neck pain, limb numbness, and muscle weakness; those associated with the cardiopulmonary system included dyspnea and chest pain; and those associated with the gastrointestinal and urological system included abdominal pain, nausea, vomiting, diarrhea, and flank pain. If a patient had more than one chief complaint, each complaint was independently categorized into the corresponding system.

### Outcomes

The outcome was positive CT findings, which were recorded in the formal reports confirmed by the radiologists. A positive CT result was confirmed when the findings or diagnoses were a newly discovered tumor, hemorrhage, fracture, soft tissue or solid organ infection, or vascular disorder (Supplementary Table 1). A negative CT result was confirmed when there were no findings or the diagnoses were static to chronic conditions, such as an old brain infarction, an old fracture, chronic inflammation, or preexisting cancer. We consulted the radiologist to reach a final decision when the diagnosis or description was vague.

### Statistical analysis

To examine consistency in the chart reviews, enough charts were selected and given to participating physicians as test samples after training. The data collection process achieved high interrater and intra-class correlation, with a kappa statistic and correlation coefficient of 0.87 and 0.93, respectively [12].

An independent data analyst who was blinded to the study design and data collection performed the statistical analysis. The normality of the continuous variables was determined by the Kolmogorov–Smirnov test. Continuous variables were presented as mean (standard deviation) or median (interquartile range). Dichotomous and categorical variables were shown as absolute sample size and percentage, respectively. The presence of different main complaints or systemic complaints between patients with positive and negative CT results was compared with the chi-squared and Fisher’s exact tests. The association between complaints and CT results was then established using univariate logistic regression. Based on logistic regression in systemic complaints, this study provided suggestions on CT with categories: reasonable (green color) if the odd ratio (OR) > 1 and the *p*-value < 0.05, equivocal (yellow color) if the *p*-value < 0.05 and might be considered (red color) if the OR < 1 and the *p*-value < 0.05.

A power analysis was performed using logistic regression z-tests via G*Power software version 3.1.9.7, establishing the required sample size. Chief complaints were analyzed as independent variables, and positive CT findings as dependent outcomes. With an alpha error set at 0.1 and power at 0.8, and assuming a 20% difference in complaints between positive and negative CT findings as clinically meaningful, the analysis determined that 131 patients were needed in each CT category to achieve statistical significance.

All other statistical analyzes were performed with Statistical Package for the Social Sciences software version 26.0 (Armonk, NY: IBM Corp.). A two-sided *p*-value less than 0.05 was considered statistically significant.

## Results

### Demographics, chief complaints, and CT in the index visit and revisit

**Fig. 1:**
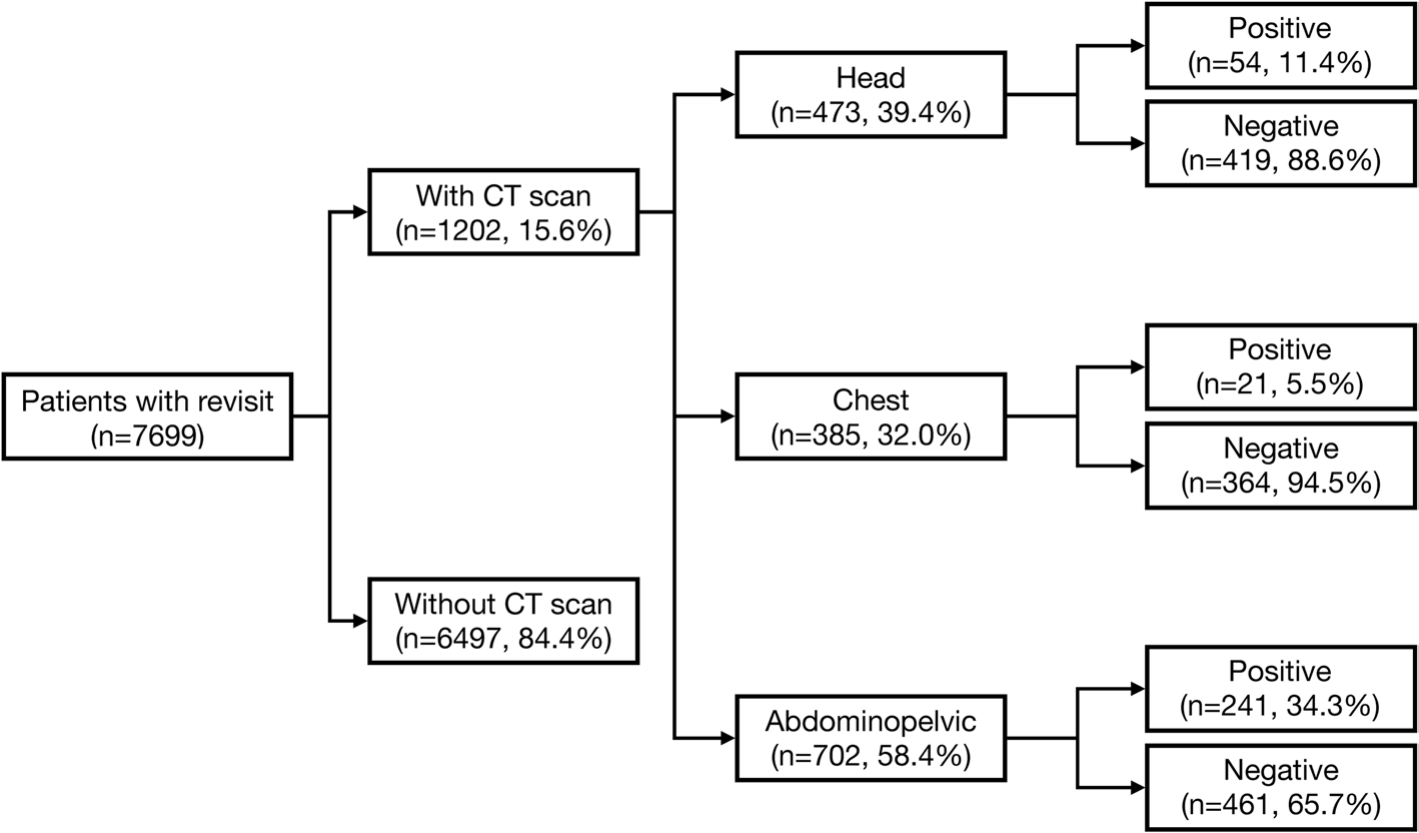
Enrollment of eligible patients with CT in the revisit

Figure 1 shows the enrollment flow chart. During the study period, 7,699 patients visited the ED after the index visit, and 1202 patients (15.6%) received CTs. Table 1 shows the demographics and CT region of the included patients. In patients who received CT on their revisit, the average age was 58 years old; men (52.0%) slightly outnumbered women (48.0%). The leading preexisting diseases were hypertension (35.7%), diabetic mellitus (21.7%), and malignancy (15.3%), followed by coronary artery disease and chronic kidney failure. Additionally, the top chief complaints at revisit included abdominal pain, dizziness, nausea and vomiting, muscles weakness, and headache. The rate of other complaints was less than 10%.

**Table 1:** The demographics, chief complaints, and CT regions

N	1202(%)
Age (years old)	58.4 ± 20.9
Males	625 (52.0)
**Pre-existing diseases**	
Hypertension	429 (35.7)
DM	261 (21.7)
CAD	120 (10.0)
CVA	51 (3.4)
Cancer	184 (15.3)
CKD	82 (6.8)
**Chief complaints**	
Headache	122 (10.1)
Dizziness	219 (18.2)
Neck pain	32 (2.7)
Chest pain	89 (7.4)
Limb numbness	28 (2.3)
Muscle weakness	148 (12.3)
Dyspnea	73 (6.1)
Abdominal pain	377 (31.4)
Nausea	113 (9.4)
Vomiting	174 (14.5)
Diarrhea	61 (5.1)
Flank pain	53 (4.4)
Chills	54 (4.5)
**Revisit with a same chief complaint**	646 (53.7)
**CT regions**	
Head	473 (39.4)
Chest	385 (32.0)
Abdominopelvic	702 (58.4)

Data are presented as number (%)

*CAD* Coronary artery disease, *CKD* chronic kidney disease, *CT* computed tomography, *CVA* cerebrovascular accident, *DM* diabetes mellitus

Among patients who had CT performed on a revisit, 473 had head CT (39.4%), 385 had chest CTs (32.0%), and 702 had abdominopelvic CTs (58.4%). On the other hand, 91 (7.6%) of the patients received CT in the index visit.

A total of 646 (53.7%) patients had same chief complaints in the index visit and the revisit. Abdominal pain (24.2%) and dizziness (10.5%) were the leading chief complaints in both visits. Electrocardiogram was performed more in the revisit (34.9% vs. 63.6%), while X-ray were administered equally in the index visit and revisit (63.8% vs. 61.8%). Around half of patients received analgesics in the index visit and revisit (51.3% vs. 51.9%). In the contrast, more patients received antibiotic treatment in the revisit than the index visit (13.6% vs. 50.0%). The detailed comparisons on each chief complaint, examination and treatment were presented in Supplementary Table 2.

### Chief complaints and CT results

Table 2 compares the CT results and chief complaints stratified by CT region. The abdominopelvic CT had the highest positive rate (34.3%), followed by the head CT (11.4%) and the chest CT (5.5%). In patients who received head CTs, the common chief complaints were dizziness (43.1%), headache (27.7%), and muscle weakness (24.7%). No significant differences were found between the chief complaints and head CT results. In patients who received chest CTs, the common chief complaints were abdominal pain (24.2%), chest pain (17.9%), and dyspnea (13.8%). Once again, no significant differences were found between the chief complaints and CT results. Furthermore, in patients who received abdominopelvic CT, the common chief complaints were abdominal pain (51.0%), vomiting (16.7%), and nausea (10.4%). The abdominopelvic CT positive rate in patients who had abdominal pain was statistically higher than in those who did not (68.8% vs 41.8%, *p* < 0.001). Inversely, the abdominopelvic CT positive rates in patients with dizziness (2.9% vs. 9.1%, *p* = 0.002), chest pain (2.5% vs 10.0%, *p* < 0.001), or dyspnea (1.3% vs. 8.7%, *p* < 0.001) were significantly lower than those of patients who did not.

**Table 2:** Comparison of chief complaints and CT result stratified by regions

	**Head**			**Chest**			**Abdominopelvic**		
	Positive	Negative	*p*	Positive	Negative	*p*	Positive	Negative	*p*
**N**	54(%)	419(%)		21(%)	364(%)		241(%)	461(%)	
**Headache**	12(22.2)	119(28.4)	0.340	0 (0)	17 (4.7)	0.613	7 (2.9)	15 (3.2)	0.812
**Dizziness**	19(35.2)	185(44.2)	0.210	3 (14.3)	30 (8.2)	0.409	7 (2.9)	42 (9.1)	0.002
**Neck pain**	8(14.8)	31(7.4)	0.062	0 (0)	9 (2.5)	1.000	0 (0)	5 (1.1)	0.172
**Chest pain**	5(9.3)	52(12.4)	0.658	7 (33.3)	62 (17.0)	0.058	6 (2.5)	46 (10.0)	< 0.001
**Limb numbness**	2(3.7)	29(6.9)	0.369	0 (0)	5 (1.4)	1.000	0 (0)	4 (1.0)	0.305
**Muscle weakness**	14(25.9)	103(24.6)	0.829	2 (9.5)	37 (10.2)	1.000	14 (5.8)	41 (8.9)	0.155
**Dyspnea**	5(9.3)	32(7.6)	0.597	1 (4.8)	52 (14.3)	0.333	3 (1.3)	40 (8.7)	< 0.001
**Abdominal pain**	2(3.7)	46(11.0)	0.146	3 (14.3)	90 (24.7)	0.431	165 (68.8)	193 (41.8)	< 0.001
**Nausea**	6(11.1)	78(18.6)	0.174	0 (0)	17 (4.7)	0.613	25 (10.4)	35 (7.6)	0.202
**Vomiting**	10(18.5)	88(21.0)	0.672	0 (0)	42 (11.5)	0.148	40 (16.7)	74 (16.0)	0.825
**Diarrhea**	0(0)	12(2.9)	0.376	2 (9.5)	17 (4.7)	0.278	18 (7.5)	33 (7.1)	0.863
**Flank pain**	2(3.7)	14(3.3)	0.703	0 (0)	17 (4.7)	0.613	14 (5.8)	36 (7.8)	0.338
**Chills**	0(0)	16(3.8)	0.236	0 (0)	33 (9.1)	0.239	15 (6.3)	24 (5.2)	0.563

Furthermore, acute cholecystitis and acute appendicitis were the predominant final diagnoses, followed by new cerebrovascular accident, intracranial hemorrhage, and soft tissue infection (see Supplementary Figure 1).

### Association between chief complaints and CT results

Table 3 shows the association between chief complaints and CT results. Patients with abdominal pain exhibited a significantly higher CT positive rate in abdominopelvic CT (OR = 3.07, 95% CI = 2.21–4.26, *p* < 0.001). On the contrary, a significantly lower CT positive rate in the abdomen was found in patients with dizziness (OR = 0.30, 95% CI = 0.13–0.68, *p* = 0.004), chest pain (OR = 0.23, 95% CI = 0.10–0.55, *p* = 0.001), and dyspnea (OR = 0.13, 95% CI = 0.04–0.44, *p* = 0.001). There was no significant association between chief complaints and CT result in head or chest CT.

**Table 3:** The association between chief complaints and positive CT result stratified by regions

	**Head**		**Chest**		**Abdominopelvic**	
**Variables**	OR (95%CI)	*p*	OR (95%CI)	*p*	OR (95%CI)	*p*
**Headache**	0.72 (0.37–1.41)	0.341	-	-	0.90 (0.36–2.23)	0.812
**Dizziness**	0.69 (0.38–1.24)	0.212	1.86 (0.52–6.66)	0.343	0.30 (0.13–0.68)	0.004
**Neck pain**	2.18 (0.94–5.02)	0.068	-	-	-	-
**Chest pain**	0.72 (0.27–1.89)	0.505	2.44 (0.94–6.28)	0.066	0.23 (0.10–0.55)	0.001
**Limb** **numbness**	0.52 (0.12–2.23)	0.377	-	-	-	-
**Muscle** **weakness**	1.07 (0.56–2.05)	0.830	0.93 (0.21–4.15)	0.925	0.64 (0.34–1.19)	0.158
**Dyspnea**	1.23 (0.46–3.32)	0.677	0.30 (0.04–2.28)	0.245	0.13 (0.04–0.44)	0.001
**Abdominal pain**	0.31 (0.07–1.32)	0.114	0.51 (0.15–1.76)	0.286	3.07 (2.21–4.26)	< 0.001
**Nausea**	0.55 (0.27–1.32)	0.180	-	-	1.42 (0.83–2.43)	0.203
**Vomiting**	0.86 (0.41–1.77)	0.672	-	-	1.05 (0.69–1.60)	0.825
**Diarrhea**	-	-	2.15 (0.46–9.98)	0.329	1.05 (0.58–1.91)	0.863
**Flank pain**	1.11 (0.25–5.03)	0.890	-	-	0.73 (0.39–1.39)	0.340
**Chills**	-	-	-	-	1.22 (0.63–2.37)	0.563

Table 4 shows the association between the chief complaints and CT results. Patients with the chief complaints in the central nervous system were less likely to receive a positive CT result on abdominopelvic CT (OR = 0.51, 95% CI = 0.31–0.82, *p* = 0.006). Patients who presented chief complaints in the cardiopulmonary system were also less likely to have a positive CT result in abdominopelvic CT (OR = 0.20, 95% CI = 0.10–0.42, *p* < 0.001). However, patients with gastrointestinal or urological chief complaints had 2.8 times the rate of positive CT results in abdominopelvic CT (OR = 2.83, 95% CI = 1.98–4.05, *p* < 0.001).

**Table 4:** The systemic complaints factors on positive or negative CT findings

	**Head**		**Chest**		**Abdominopelvic**	
Variables	OR (95%CI)	*p*	OR (95%CI)	*p*	OR (95%CI)	*p*
Chief complaints						
CNS	0.65 (0.36–1.16)	0.143	0.97 (0.32–2.98)	0.959	0.51 (0.31–0.82)	0.006
Cardiopulmonary	1.37 (0.55–3.43)	0.499	1.65 (0.66–4.09)	0.283	0.20 (0.10–0.42)	< 0.001
GI or urological	0.68 (0.32–1.45)	0.318	0.38 (0.13–1.16)	0.088	2.83 (1.98–4.05)	< 0.001

*CNS* Central nervous system, *GI* gastrointestinal

**Fig. 2:**
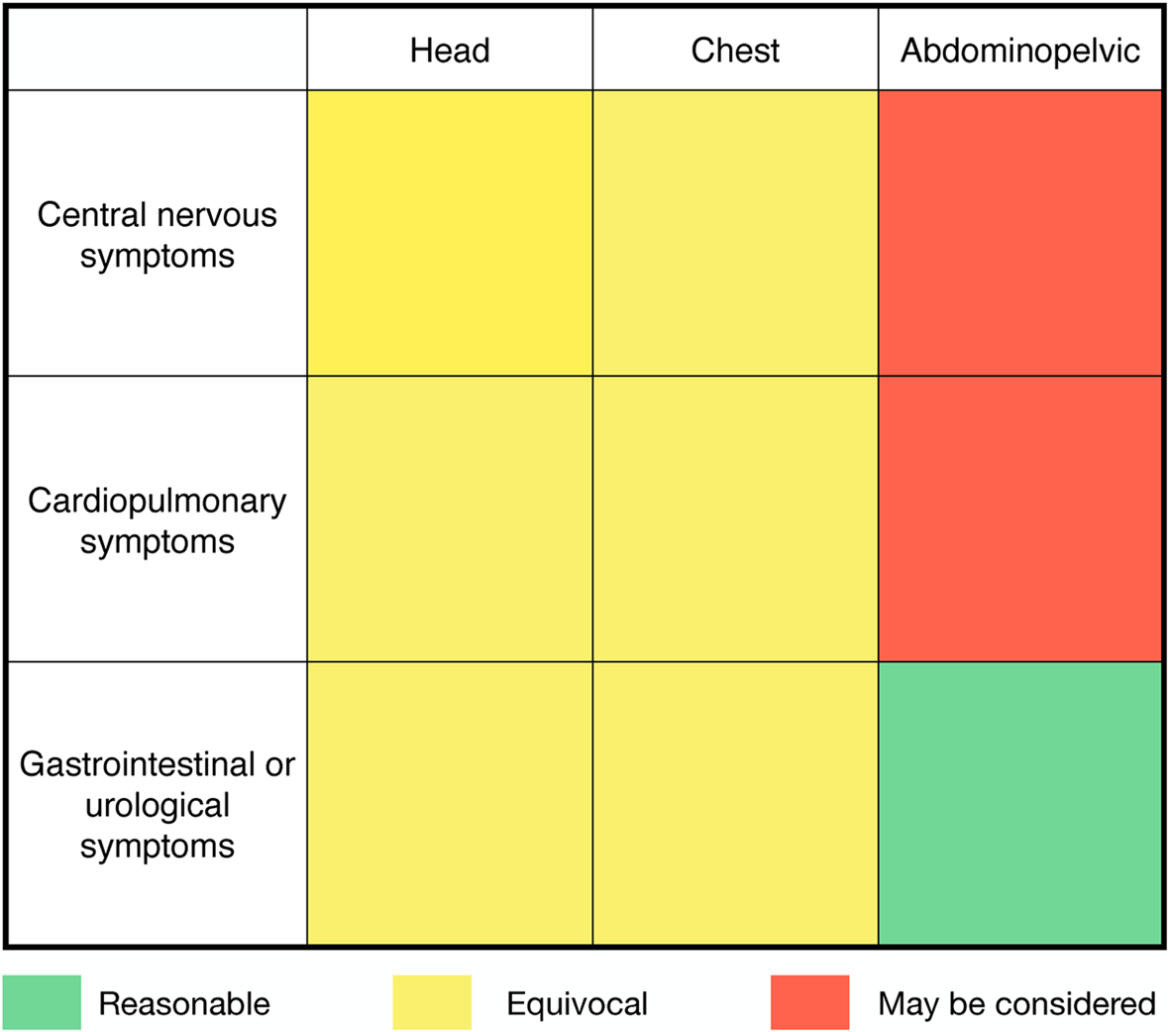
Suggestion on CT utility according to systematic complaints

Figure 2 shows a summary of this study’s results. Patients who presented gastrointestinal or urological symptoms on the re-examines and received abdominopelvic CT were considered reasonable in terms of CT category. A higher positive CT rate could be expected compared with those who presented with central nervous system symptoms or cardiopulmonary symptoms in revisit. Patients who presented each system-based symptom and received a corresponding CT were considered equivocal.

## Discussion

In this study, we investigated the potential association between chief complaints and positive CT results in patients who returned to the ED after their index visit. The overall positive rate was < 40% among all CTs performed. In patients who received head and chest CT scans, the percent with positive findings did not differ between the complaints studied. In particular, abdominopelvic CT had the highest positive rate. In patients who had abdominopelvic CT scans, those with abdominal pain had more frequent positive scans compared to those without abdominal pain. Those with dizziness, chest pain, and dyspnea had fewer positive abdominopelvic CTs compared to those without these complaints.

Pain is a common complaint that informs ED physicians’ decisions of emergency physicians to perform a CT to identify a potential emergency [13–15]. The current study indicated that among all chief complaints, abdominal pain is the most valuable indicator for performing a CT. One previous study has indicated that abdominal pain can be correctly addressed when history and physical examination are obtained properly [16]; therefore, this condition may not be completely ideal in a real clinical setting. Furthermore, patients who presented with neurological or cardiopulmonary symptoms on return were less likely to have a positive CT result than in the abdominal region. Modification of workplace culture or defensive medicine may be the cause of this high-frequency use [17].

Admittedly, both positive and negative CT findings could be clinically significant. Based on the guidelines of the British Society of Cardiovascular Imaging, initial administration of a chest CT alone is recommended in patients with low to intermediate risk without abdominal pain or symptoms of the lower extremities of acute aortic syndrome [18]. In contrast, patients with acute chest pain reasonably receive a chest CT to exclude acute aortic syndrome or pulmonary embolism [19]. However, our results indicated that the chief complaint of chest pain was not associated with an increased CT positive rate in the chest region. The low overall yield rate of chest CT (5.5%) may be a concern. Furthermore, we found that chest CT was frequently in patients with abdominal pain, which was sometimes compatible with the clinical setting. An explanation may be the need for a complete aorta evaluation when acute aortic syndrome was not fully excluded.

Moreover, compound chief complaints made the diagnosis difficult because the existing criteria had great sensitivity but poor specificity. We included central nervous system-related complaints that combined several corresponding chief complaints; however, the association between system-based chief complaints and positive CT results in the head remained non-significant. Muscle weakness, often mimicking hemiparesis or hemiparalysis according to the patients, was a frequent complaint in those who received head CT. However, the positive and negative head CT rates were similar (Table 2). These equivocal findings may have originated from the clinical system rather than the individual patient. For example, a routine head CT scan is required to exclude potential intracranial hemorrhage in suspected stroke patients [20]. In addition, other systems factors might play a crucial role in the physician’s decision to order a head CT [21]. Further studies are required to determine the association between other complaints and head CT results.

In this study, the data were collected directly from the medical records after independent physician reviews rather than from a preprocessed, structured database. Also, CT findings and positive results were determined by formal reports rather than using the International Classification of Diseases code, which prevented information bias [22]. However, this study has several limitations. First, data were retrospectively obtained from a single hospital. Therefore, the results may not be applicable to other hospitals with different populations of patients. Only physicians from a single center were included, which could also cause bias. Second, the overall CT administration in the ED revisit was low (15.6%), which might cause selection bias to the analysis and limit the generalizability to the broader population of ED revisit. Further prospective studies should be performed to validate the current results. Third, due to the study’s retrospective nature, some complaints might have been missed because the ED physician often records the most important chief complaints alone. A multicenter study is needed to eliminate the biases mentioned above.

## Conclusion

This study provided three-level suggestions on the utility of CTs for different system-based chief complaints. The use of abdominopelvic CT might be reasonable if the patient has abdominal pain or complaints in the gastrointestinal system. However, the use of abdominopelvic CT in patients with complaints from other systems might not yield new findings. The use of head or chest CT for other complaints is equivocal, depending on the condition.

### Supplementary Information

Supplementary Material 1.

Supplementary Material 2.

## Data Availability

The raw data is available on request to the corresponding author.
