# Supplementary material for: Chief complaints and computed tomography results in the emergency department: a three-year retrospective cohort study
Source: BMC Emerg Med. 2024 May 20;24:87. doi: 10.1186/s12873-024-01003-z (PMC11103846; doi:10.1186/s12873-024-01003-z)
Supplement: Supplementary file 1 — Supplementary Material 1. [file 12873_2024_1003_MOESM1_ESM.docx]

| Supplementary Table 1. The definition of CT positive result | |
| --- | --- |
| Head CT | Newly discovered tumor, intracranial hemorrhage, new cerebrovascular accident, soft tissue infection or abscess, fracture, encephalopathy |
| Chest CT | Newly discovered tumor, soft tissue infection or abscess, fracture, acute coronary syndrome, aortic aneurysm, pulmonary embolism, empyema, pulmonary hemorrhage, pneumothorax, pneumomediastinum |
| Abdominopelvic CT | Newly discovered tumor, soft tissue infection or abscess, fracture, aortic aneurysm, solid organ abscess, cholecystitis, biliary tract obstruction, pancreatitis, appendicitis, diverticulitis, ischemic bowel, intussusception, acute pyelonephritis, obstructive nephropathy, ovarian torsion, tubo-ovarian abscess, pneumoperitoneum, other intra-abdominal infection |

CT: computed tomography

| Supplementary Table 2. Comparisons of chief complaints, examinations and treatment between index visit and revisit (N = 1,202) | | | |
| --- | --- | --- | --- |
|  | **Index visit** | **Revisit** | **Both visits** |
| **Chief complaints** |  |  |  |
| Headache | 106 (8.8) | 122 (10.1) | 57 (4.7) |
| Dizziness | 192 (16.0) | 219 (18.2) | 126 (10.5) |
| Neck pain | 38 (3.2) | 32 (2.7) | 17 (1.4) |
| Chest pain | 101 (8.4) | 89 (7.4) | 38 (3.2) |
| Limb numbness | 17 (1.4) | 28 (2.3) | 8 (0.7) |
| Muscle weakness | 86 (7.2) | 148 (12.3) | 48 (4.0) |
| Dyspnea | 67 (5.6) | 73 (6.1) | 24 (2.0) |
| Abdominal pain | 384 (31.9) | 377 (31.4) | 291 (24.2) |
| Nausea | 123 (10.2) | 113 (9.4) | 37 (3.1) |
| Vomiting | 182 (15.1) | 174 (14.5) | 77 (6.4) |
| Diarrhea | 67 (5.6) | 61 (5.1) | 32 (2.7) |
| Flank pain | 67 (5.6) | 53 (4.4) | 35 (2.9) |
| Chills | 47 (3.9) | 54 (4.5) | 20 (1.7) |
| **Examinations** |  |  |  |
| Electrocardiogram | 420 (34.9) | 765 (63.6) | 271 (22.5) |
| X-ray | 767 (63.8) | 743 (61.8) | 465 (38.7) |
| **Treatments** |  |  |  |
| Analgesics | 617 (51.3) | 624 (51.9) | 432 (35.9) |
| Antibiotics | 163 (13.6) | 601 (50.0) | 137 (11.4) |
| Data are presented as number (%). CT: computed tomography | | | |
