# Supplementary figures and images for: Chief complaints and computed tomography results in the emergency department: a three-year retrospective cohort study
Source: BMC Emerg Med. 2024 May 20;24:87. doi: 10.1186/s12873-024-01003-z (PMC11103846; doi:10.1186/s12873-024-01003-z)

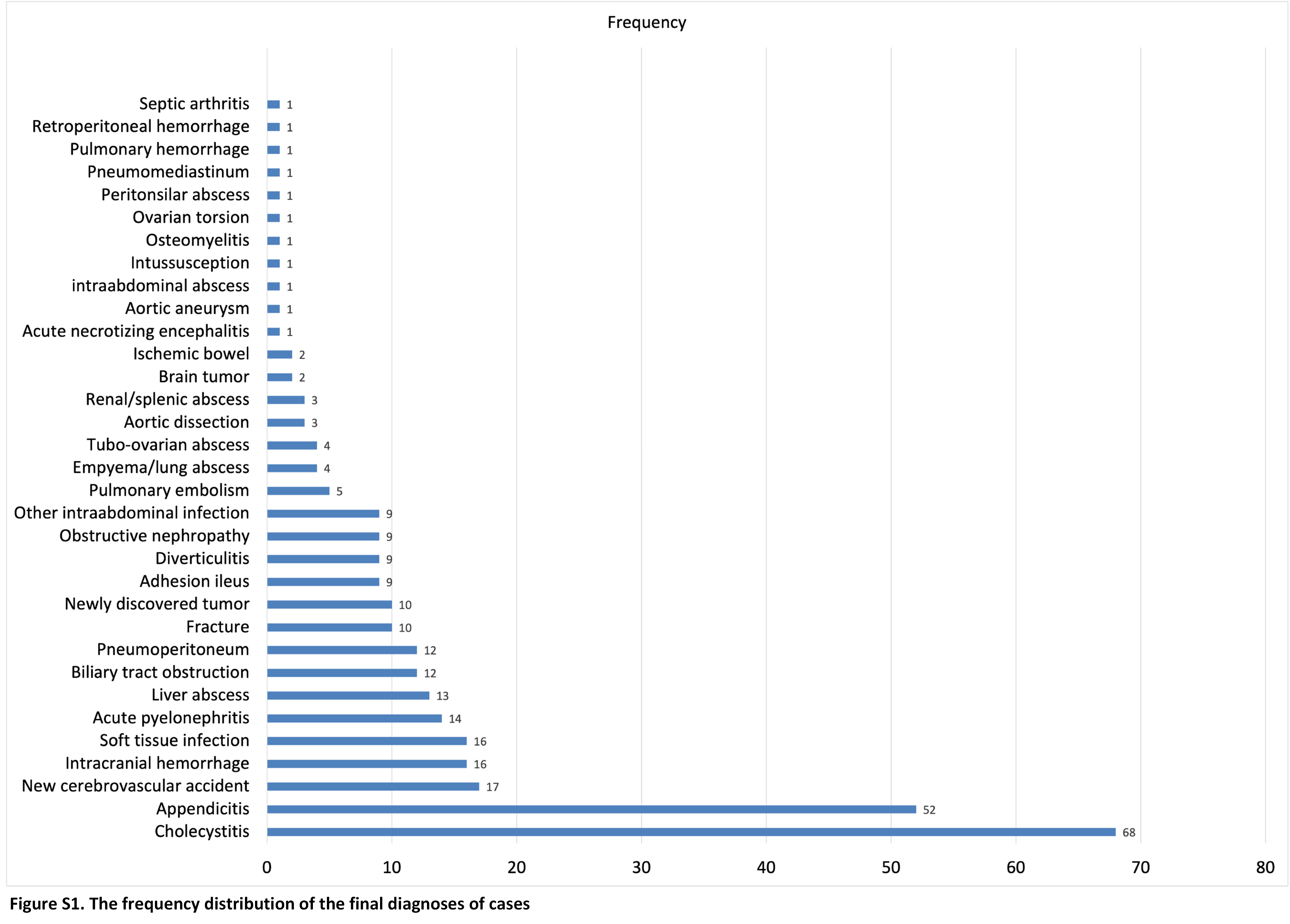

Supplement: Supplementary file 2 — Supplementary Material 2. [file 12873_2024_1003_MOESM2_ESM.png]
